# Impact of Glucagon-Like Peptide-1 Receptor Agonists on Hip Arthroplasty Outcomes: A Systematic Review and Meta-Analysis

**DOI:** 10.7759/cureus.96279

**Published:** 2025-11-07

**Authors:** Haroon Zaffar, Mohammed A Imran, Sulaiman Hussain, Farwah Rushd

**Affiliations:** 1 Surgery, University of Lancashire, Preston, GBR; 2 Orthopaedics and Traumatology, Royal Alexandra Hospital, Paisley, GBR; 3 Trauma and Orthopaedics, Stoke Mandeville Hospital, Aylesbury, GBR; 4 College of Medicine, University of Glasgow, Glasgow, GBR

**Keywords:** 90-day readmission, glucagon-like peptide-1 receptor agonists, post-operative complications, semaglutide, total hip arthroplasty

## Abstract

Obesity and diabetes are common among patients undergoing total hip arthroplasty (THA) and are associated with adverse outcomes. Glucagon-like peptide 1 receptor agonists (GLP-1 RAs) were originally developed for glycaemic control but have recently been approved for weight reduction. Given these dual metabolic effects, their perioperative use is of growing interest. Despite this, the impact of GLP-1 RAs on post-operative outcomes remains underexplored. This systematic review and meta-analysis aim to address this evidence gap.

A literature search was conducted in MEDLINE, PubMed, Embase, and CENTRAL from inception to 1st June 2025. Studies comparing outcomes between GLP-1 RA users and non-users in adults (≥18 years) undergoing primary THA were included. Primary outcomes included medical and surgical complications. Secondary outcomes included hospital-related measures such as 90-day readmissions and length of stay. Risk of bias was assessed using the Risk of Bias in Non-randomized Studies of Interventions (ROBINS-I) tool, and the certainty of evidence was evaluated using the Grading of Recommendations, Assessment, Development and Evaluations (GRADE) approach.

Six retrospective cohort studies, all conducted in the United States, met the inclusion criteria and included 11,869 GLP-1 RA users and 22,777 controls. GLP-1 RAs use was associated with a statistically significant reduction in 90-day readmission rates (odds ratio (OR) 0.81, 95% confidence interval (CI) 0.69-0.94, p = 0.007; I^2^ = 39%) and short-term revision surgery (OR 0.70, 95% CI 0.52-0.94, p = 0.02; I^2^ = 18%). No significant differences were observed for other medical or surgical complications.

GLP-1 RAs were associated with reduced short-term revision rates and 90-day readmissions following THA. However, as only retrospective studies were identified, high-quality prospective studies are needed to confirm these findings.

## Introduction and background

Total hip arthroplasty (THA) is among the most performed elective orthopaedic procedures [1]. According to the 2024 National Joint Registry Report, demand is increasing with 108,558 primary THAs being performed across the United Kingdom in 2023 - the highest figure to date [2]. Similar trends are seen internationally, with annual counts being expected to nearly double to 850,000 by 2030 in the United States (US) [3]. The primary indication for THA is end-stage osteoarthritis, which is highly prevalent in patients with obesity and diabetes mellitus (DM) [1,4,5].

Both obesity and DM are established risk factors for adverse outcomes following THA [6,7]. A recent meta-analysis reported an almost fourfold increase in the risk of periprosthetic joint infection (PJI) in morbidly obese patients as well as higher rates of readmission, revision surgery, and superficial infection [6]. Similarly, Chun et al. reported that patients with DM undergoing THA experienced higher rates of surgical site infections (SSIs) and periprosthetic fractures (PPFs) [7]. Due to the rising prevalence of both comorbidities, optimisation of weight and glycaemic control is becoming increasingly crucial to reduce the risk of post-operative complications [8,9].

Various strategies have been investigated to aid pre-operative weight loss, including dietary changes, lifestyle modification, and bariatric surgery [10]. Digital interventions show good engagement but modest weight loss, whereas bariatric surgery achieves sustained BMI reduction but carries risks of nutritional deficiency and adverse THA outcomes [11-13].

These limitations, along with growing public interest, have led to the popularisation of pharmaceutical alternatives such as glucagon-like peptide-1 receptor agonists (GLP-1 RAs) [14]. Originally developed for glycaemic control in DM, these agents have recently been approved for weight loss purposes in the US [15,16]. GLP-1 RAs act by mimicking endogenous incretin hormones to enhance insulin secretion and improve glycaemic control [17]. They also slow down gastric emptying and suppress appetite, which contributes to weight reduction [17]. These physiological effects may positively influence perioperative outcomes, particularly in patients undergoing major procedures such as THA, where obesity and diabetes are known risk factors for complications [18].

Despite emerging evidence, the impact of GLP-1 RAs on outcomes following THA remains underexplored. This systematic review and meta-analysis aim to address this gap by evaluating if GLP-1 RA use is associated with improved post-operative outcomes in patients undergoing THA.

## Review

Methods

This review adhered to the Methodological Expectations of Cochrane Intervention Reviews (MECIR) standards and followed the Preferred Reporting Items for Systematic Reviews and Meta-Analyses (PRISMA) 2020 guidelines [19,20]. It was registered with PROSPERO (CRD4202510522170). Institutional Review Board approval was not required for this study, as it was a systematic review and meta-analysis of previously published data.

Eligibility Criteria

Studies were selected using the PICO (Population, Intervention, Comparator, Outcome) framework. Adults (≥18 years) undergoing primary THA were included; patients undergoing revision surgery were excluded. The intervention was the use of GLP-1 RA, regardless of indication (obesity or DM). Comparators were patients undergoing primary THA without GLP-1 RA use.

Primary outcomes assessed medical and surgical complications. Medical complications included deep vein thrombosis (DVT), pulmonary embolism (PE), acute kidney injury (AKI) and pneumonia. Surgical complications included SSI, wound dehiscence, PJI, PPF and revision surgery. Secondary outcomes were 90-day hospital readmissions and length of stay (LOS).

Randomised, non-randomised and observational studies published in English were eligible. Excluded were case reports, abstracts without full-text, letters and non-peer-reviewed publications.

Search Strategy

A comprehensive search was performed in MEDLINE, PubMed, Embase, and CENTRAL, from inception to 1st June 2025. Grey literature and clinical trial registries were not searched. Search strategy included terms relating to THA, GLP-1 RA and relevant outcomes. The full search strategy is available in Appendix A.

Selection Process

Three reviewers independently screened titles, abstracts and full texts against the eligibility criteria. Discrepancies were resolved by discussion.

Data Collection Process

Two reviewers independently extracted data into a Microsoft Excel sheet (Microsoft Corp., Redmond, WA, USA), including study characteristics, demographics, intervention details and outcomes stratified by timing (short-term: ≤90 days, long-term: ≥1 year). Percentages were converted to raw counts where possible. Discrepancies were resolved through discussion and/or author contact, where possible. We addressed missing data by contacting study authors where possible to obtain clarification.

Risk of Bias Assessment

Risk of bias was assessed using the Cochrane Risk of Bias 2 (RoB-2) tool for randomised trials and the Risk Of Bias In Non-randomised Studies of Interventions (ROBINS-I) tool for observational studies [21,22]. Two reviewers independently assessed each domain and graded the overall risk of bias as low, moderate, serious or critical.

Reporting Bias Assessment

Reporting bias was assessed by comparing study protocols to published outcomes. Funnel plots and Egger’s test were employed for outcomes consisting of data from 10 or more studies. No formal assessment was undertaken when fewer than 10 studies were available.

Certainty Assessment

Certainty of evidence was assessed using the GRADE (Grading of Recommendations, Assessment, Development and Evaluation) tool across five domains: risk of bias, inconsistency, indirectness, imprecision and publication bias [23]. Two reviewers independently rated the evidence.

Data Synthesis and Statistical Analysis

Meta-analyses were performed using ReviewManager software (Version 5.4.1; The Cochrane Collaboration, London, England, UK). Dichotomous outcomes were pooled as odds ratios (ORs) with 95% confidence intervals (CIs) using a random-effects model. Between-study variance was estimated using the DerSimonian and Laird method. Heterogeneity was assessed using the Chi^2^ test and I^2^ statistic as per the Cochrane Handbook [24]. P-value <0.05 was considered statistically significant. Sensitivity analyses accounted for unclear events, and pre-specified subgroup analyses were conducted based on GLP-1 RA type and diabetes status wherever possible. When meta-analysis was not feasible, narrative synthesis was used.

Results

Search Results

The search identified 288 records. After removing 96 duplicates and three non-English records, 189 records were screened by title and abstract, leading to the exclusion of 174 irrelevant records. Fifteen full-text articles were assessed.

**Figure 1:**
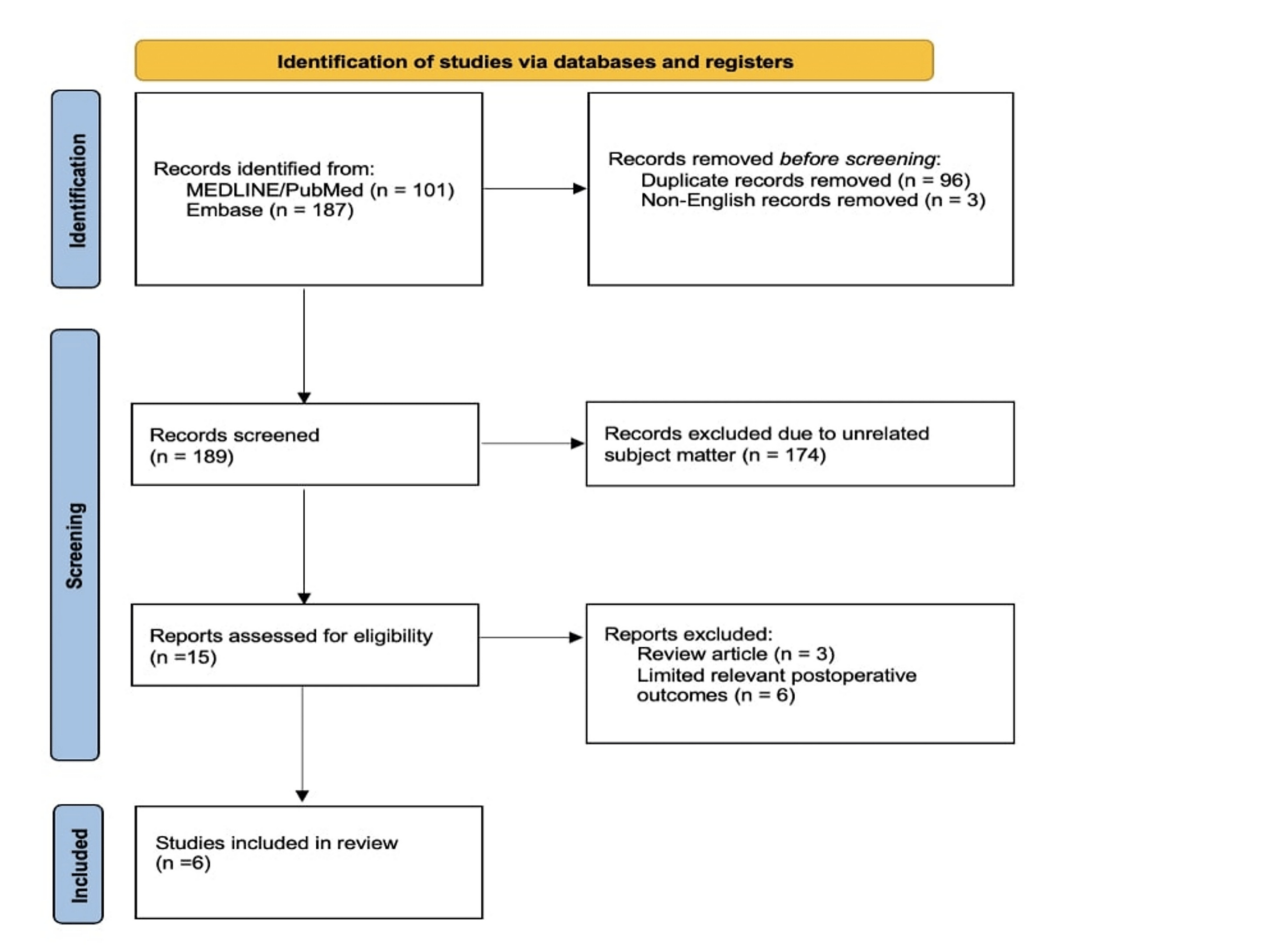
Preferred Reporting Items for Systematic Reviews and Meta-Analyses (PRISMA) flow diagram of study search and inclusion.

Six studies met the inclusion criteria and were included in the qualitative and quantitative synthesis. Two full-text articles were excluded after a detailed review. Mahmoud et al. focused solely on perioperative aspiration risk in diabetic patients, without reporting surgical or medical complications [25]. Magaldi et al. assessed limited medical complications in a small cohort (n=66) [26]. The study selection process is summarised in the PRISMA 2020 flow diagram (Figure 1).

Characteristics of Included Studies

All six included studies were matched retrospective cohorts from the US, totalling 11,869 GLP-1 RA users and 22,777 controls [27-32]. Mean age ranged from 57 to 63.5 years, with men comprising 42% to 58% of participants. All studies assessed post-operative outcomes following THA with varying follow-up. Table 1 summarises these characteristics.

**Table 1:** Study characteristics. ^a^ In Magruder et al. [31], the number (%) of patients in each age category in the GLP-1 RA versus control cohorts were as follows: 45-49 years, 58 (3.5) versus 267 (3.4); 50-54 years, 163 (9.9) versus 749 (9.6); 55-59 years, 350 (21.2) versus 1,661 (21.3); 60-64 years, 442 (26.7) versus 2,101 (26.9); 65-69 years, 362 (21.9) versus 1,739 (22.3); 70-74 years, 189 (11.4) versus 904 (11.6); and 75-79 years, 77 (4.7) versus 349 (4.5). No patients were reported in age groups below 45 years or above 79 years. GLP-1 RA: Glucagon-like peptide 1 receptor agonist

Author and year	GLP-1 (n)	Control (n)	Mean age (year)		Men, N (%)		GLP-1 RA agent	Outcomes assessed
			GLP-1	Control	GLP-1	Control		
Buddhiraju 2024 [27]	1044	1044	63.3	63.5	442 (42.3)	427 (40.9)	NR	90 days medical, 90 days revision
Heo 2024 [28]	812	3248	61	60	473 (58.3)	1871 (57.6)	NR	90 days medical, 1 year surgical
Kim 2024 [29]	771	3084	62.1	62.1	364 (47.2)	1453 (47.1)	Exenatide (including microsphere formulation), semaglutide, dulaglutide, liraglutide.	90 days medical and surgical, 2 years surgical
Levidy 2025 [30]	2244	2244	NR		NR		Liraglutide, pramlintide, tirzepatide, semaglutide, lixisenatide, Sulaglutide	90 days and 1 year surgical outcomes
Magruder 2023 [31]	1653	7812	^a^	^a^	866 (52.4)	4076 (52.2)	Semaglutide	90 days medical, 2 years surgical
Verhey 2024 [32]	5345	5345	57	57	1671 (45.5)	1656 (31)	Liraglutide, semaglutide, dulaglutide, exenatide, lixisenatide	90 days medical, 2 years surgical

Medical complications

Deep Vein Thrombosis (DVT)

**Figure 2:**
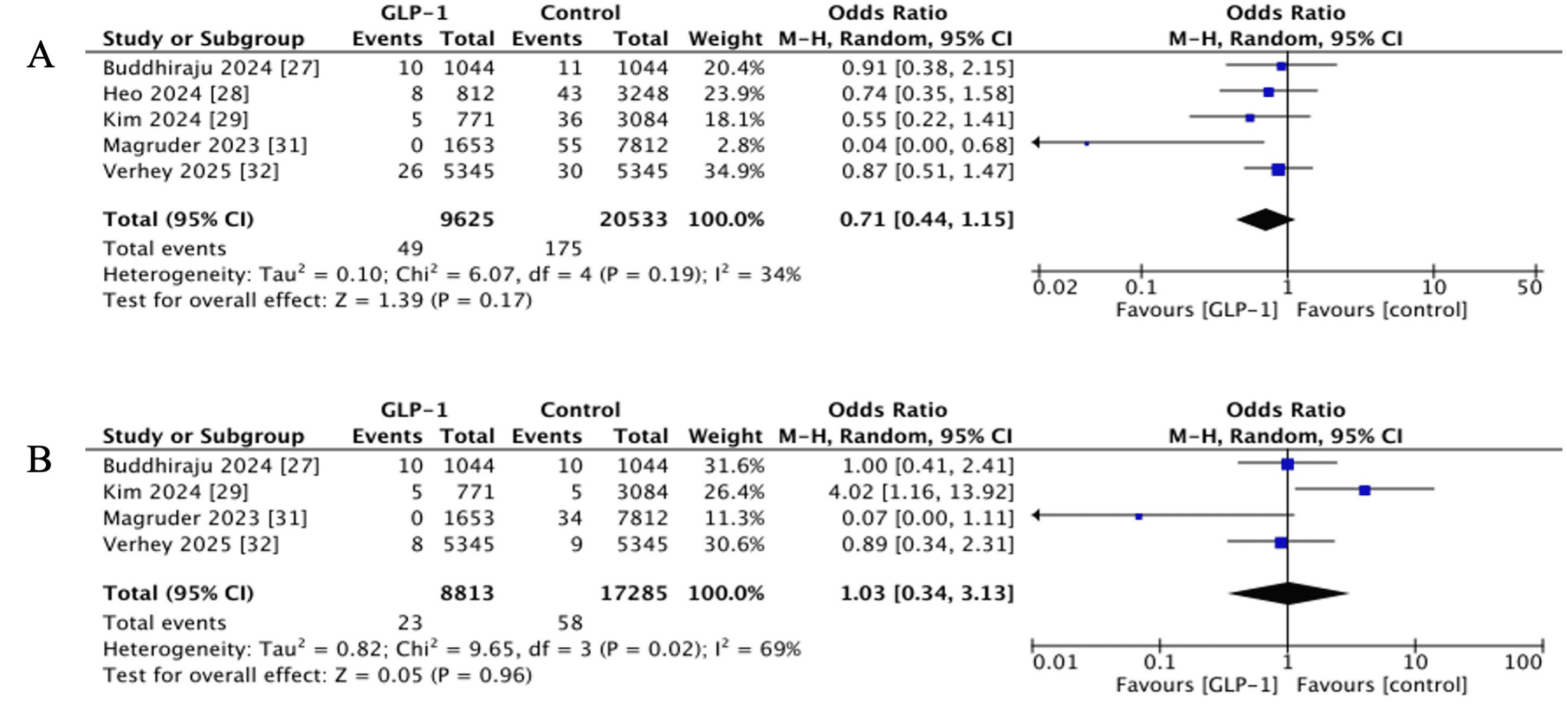
Forest plots showing pooled effect estimates for: (A) Deep vein thrombosis, (B) pulmonary embolism. GLP-1: Glucagon-like peptide 1 References [27-29,31,32]

Five studies evaluated the cases of DVT within 90 days post-operatively [27-29,31,32]. The pooled OR indicated no statistically significant difference between GLP-1 RA users and the control (OR 0.71, 95% CI 0.44-1.15, p = 0.17; I^2^ = 34%), although most studies reported a direction of effect favouring GLP-1 users (Figure 2).

Pulmonary Embolism (PE)

Four studies reported 90-day PE events [27,29,31,32]. No notable difference was observed (OR 1.03, 95% CI 0.34-3.13, p = 0.96; I^2^ = 69%), with high heterogeneity in effect estimates (Figure 2).

Pneumonia

**Figure 3:**
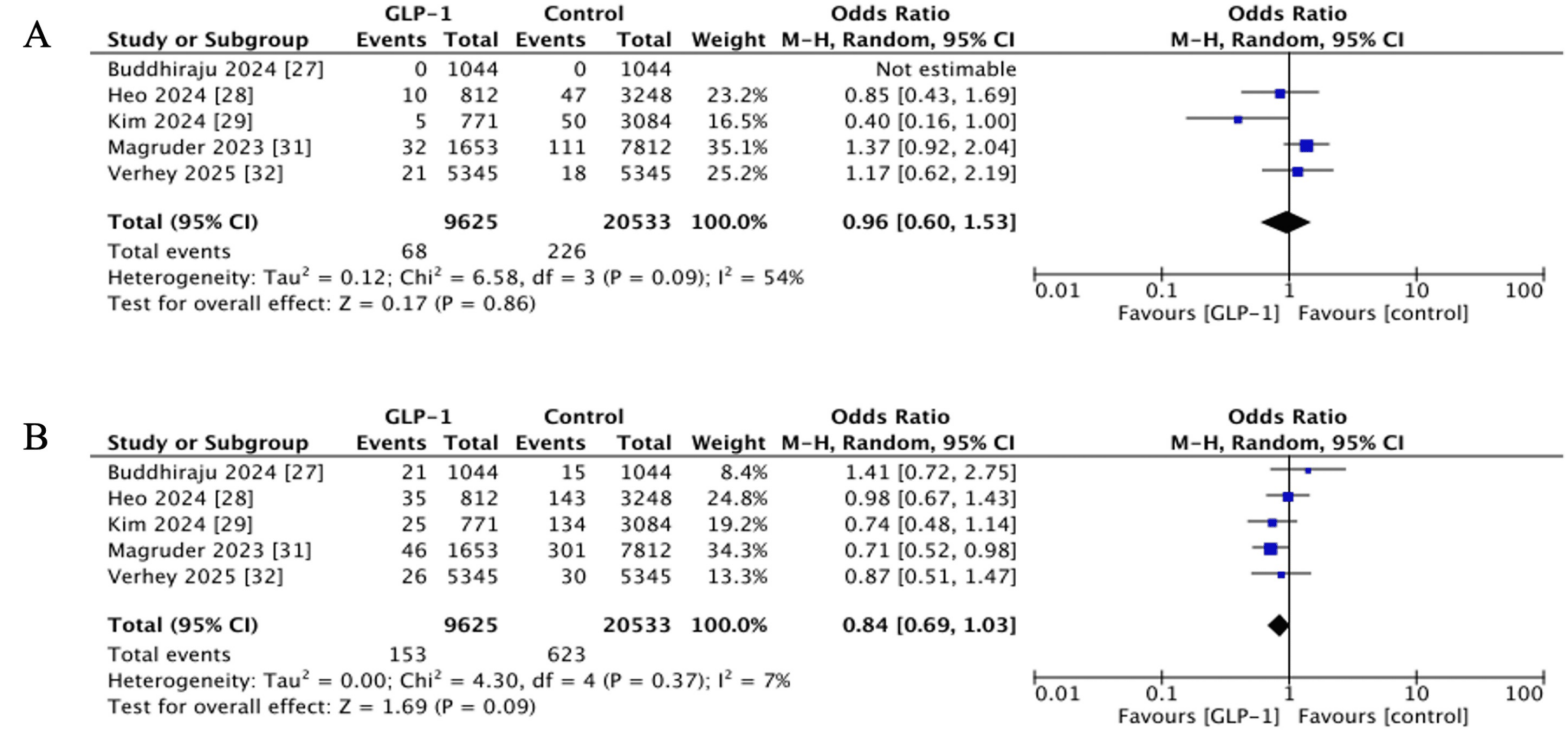
Forest plots showing pooled effect estimates for: (A) Pneumonia, (B) acute kidney injury. GLP-1: Glucagon-like peptide 1 References [27-29,31,32]

Five studies examined pneumonia occurring within 90 days of surgery [28,29,31,32]; one paper [27] was excluded due to zero events. Combined analysis demonstrated a non-significant effect (OR 0.96, 95% CI 0.60-1.53, p = 0.86; I^2^ = 54%) (Figure 3).

Acute Kidney Injury (AKI)

AKI within 90 days was reported in five studies [27-29,31,32]. The pooled estimate suggested a trend towards reduced risk among GLP-1 RA users, though this did not reach statistical significance (OR 0.84, 95% CI 0.69-1.03, p = 0.09; I^2^ = 7%) (Figure 3).

Surgical complications

Surgical Site Infection (SSI)

**Figure 4:**
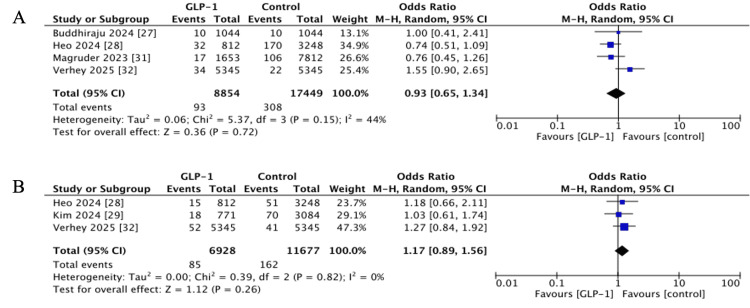
Forest plots showing pooled effect estimates for: (A) Surgical site infection, (B) wound dehiscence. GLP-1: Glucagon-like peptide 1 References [27,28,31,32]

SSI within 90 days was assessed in four studies [27,28,31,32]. The pooled OR showed no significant difference (OR 0.93, 95% CI 0.65-1.34, p = 0.72; I^2^ = 44%) between the two groups (Figure 4).

Wound Dehiscence

Wound dehiscence was reported in three studies [28,29,32]. The pooled OR was 1.17 (95% CI 0.89-1.56, p = 0.26; I^2^ = 0%), indicating no statistically significant difference between GLP-1 RA users and controls. However, all studies showed a direction of effect towards slightly increased odds (Figure 4).

Periprosthetic Joint Infection (PJI)

**Figure 5:**
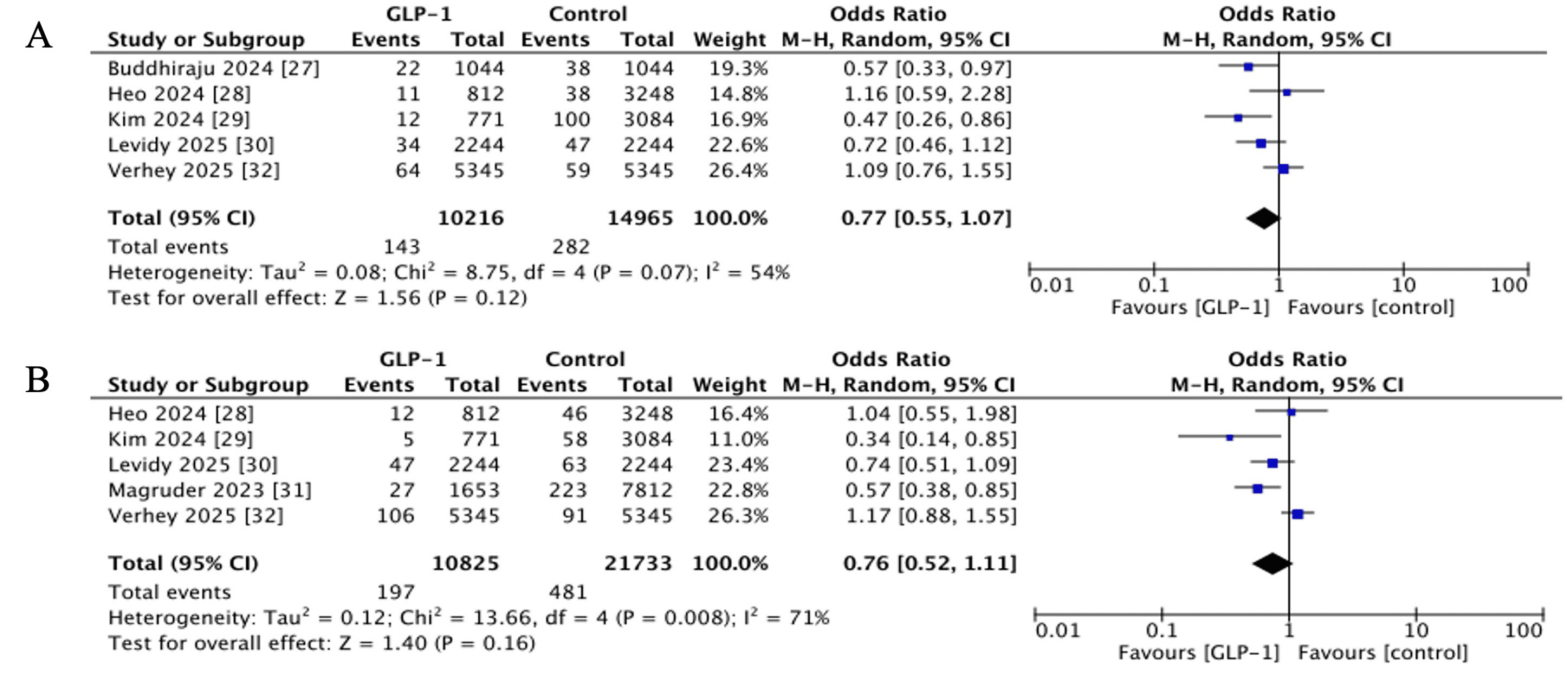
Forest plots showing pooled effect estimates for periprosthetic joint infection: (A) Short-term, (B) long-term GLP-1: Glucagon-like peptide 1 References [27-32]

Short-term PJI outcomes were reported in five studies [27-30,32], with a pooled OR of 0.77 (95% CI 0.55-1.07, p = 0.12; I^2^ = 54%). Long-term outcomes, reported in five studies with follow-up duration ranging from one to two years [28-32], also showed no significant difference (OR 0.76, 95% CI 0.52-1.11, p = 0.16; I^2^ = 71%) (Figure 5).

Periprosthetic Fracture (PPF)

**Figure 6:**
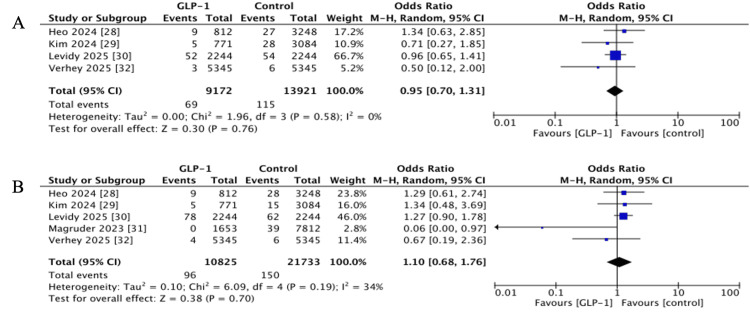
Forest plots showing pooled effect estimates for periprosthetic fracture: (A) Short-term, (B) long-term. GLP-1: Glucagon-like peptide 1 References [28-32]

Four studies evaluated short-term PPF [28-30,32], with no significant association observed (OR 0.95, 95% CI 0.70-1.31, p = 0.76; I^2^ = 0%). Long-term PPF were evaluated across five studies with one to two years follow-up [28-32], and again showed no significant difference (OR 1.10, 95% CI 0.68-1.76, p = 0.70; I^2^ = 34%) (Figure 6).

Revision Surgery

**Figure 7:**
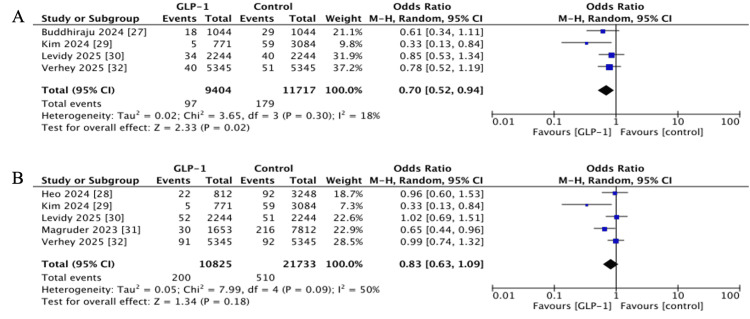
Forest plots showing pooled effect estimates for Revision: (A) Short-term, (B) long-term. GLP-1: Glucagon-like peptide 1 References [27-32]

Short-term revision cases were pooled from four studies [27,29,30,32], with a statistically significant reduction in risk among GLP-1 RA users (OR 0.70, 95% CI 0.52-0.94, p = 0.02; I^2^ = 18%). However, long-term data from five studies with one to two years of follow-up did not show a significant effect (OR 0.83, 95% CI 0.63-1.09, p = 0.18; I^2^ = 50%) (Figure 7) [28-32].

Secondary outcomes

90-Day Readmission

**Figure 8:**
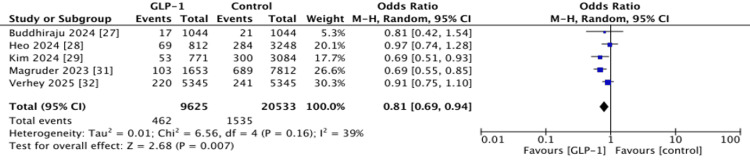
Forest plots showing pooled effect estimates for 90-day readmission. GLP-1: Glucagon-like peptide 1 References [27-29,31,32]

Five studies reported on 90-day hospital readmission rates. GLP-1 RA use was associated with a statistically significant reduction in the odds of readmission compared to controls (OR 0.81, 95% CI 0.69-0.94; p = 0.007) [27-29,31,32]. Heterogeneity was moderate (I^2^ = 39%) (Figure 8).

Length of Stay (LOS)

LOS reporting varied across studies and was not suitable for meta-analysis. Magruder et al. reported a non-significant reduction in mean LOS in the GLP-1 RA group (2.7 vs. 2.9 days) [31]. Kim et al. reported lower LOS with GLP-1 RA use (2.2 days, SD 1.4 vs. 3.1 days, SD 4.0; p = 0.01) [29]. Heo et al. reported fewer patients with LOS > 3 days in the GLP-1 RA group (24.4% vs 28.5%; p = 0.01) [28].

Risk of Bias Assessment

**Figure 9:**
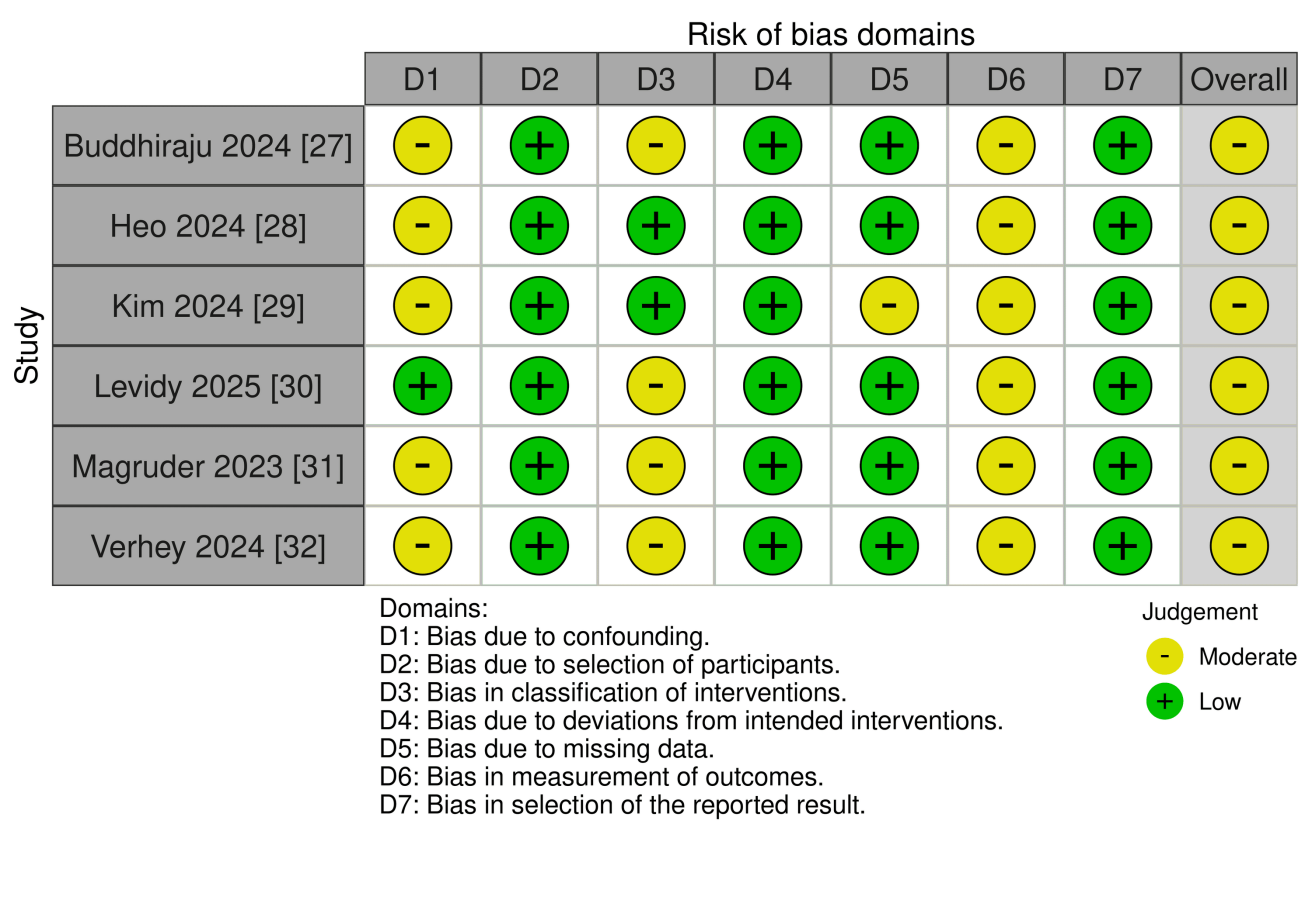
Risk of bias summary using the ROBINS-I tool for all included studies. ROBINS-I: Risk of Bias in Non-randomized Studies of Intervention References [27-32]

Six studies were assessed as having a moderate overall risk of bias, mainly due to confounding and intervention misclassification. A summary of assessments is shown in Figure 9, with detailed information shown in Appendix B.

Certainty of Evidence

Seven outcomes were assessed as having very low certainty using GRADE, covering medical, surgical and hospital-related outcomes. Downgrades were due to study design, imprecision and inconsistency. Full ratings are presented in Table 2.

**Table 2:** Summary of findings table. CI: confidence interval; OR: odds ratio; GLP-1: glucagon-like peptide 1 ^a^ All studies had a moderate risk of bias; downgraded by one level. ^b^ Rated as serious due to the pooled findings being sensitive to assumptions made about unclear data in a key study (Kim et al. 2024). Although event counts of 5 and 10 yielded a significance was lost when the lowest possible event count of 1 was assumed. ^c^ Rated as serious due to substantial heterogeneity (I^2^ ≥ 50%) and inconsistent effects across studies. ^d^ Rated as serious due to confidence interval crossing the line of no effect (i.e., including 1.0).

Outcome	Participants (studies) follow-up	Risk of bias	Inconsistency	Indirectness	Imprecision	Publication bias	Overall certainty of evidence	Study event rates (%) with control	Study event rates (%) with GLP-1	Relative effect (95% CI)
Short-term revision	21,121 (4 non-randomised studies)	seriousᵃ	not serious	not serious	seriousᵇ	none	very low	179/11,717 (1.5%)	97/9404 (1.0%)	OR 0.70 (0.52 to 0.94)
Long-term revision	32,558 (5 non-randomised studies)	seriousᵃ	serious^ c^	not serious	serious^d^	none	very low	510/21,733 (2.3%)	200/10,825 (1.8%)	OR 0.83 (0.63 to 1.09)
Short-term periprosthetic joint infection	23,093 (4 non-randomised studies)	seriousᵃ	not serious	not serious	serious^d^	none	very low	244/13,921 (1.8%)	121/9172 (1.3%)	OR 0.82 (0.56 to 1.20)
Pulmonary embolism	26,098 (4 non-randomised studies)	seriousᵃ	serious^ c^	not serious	serious^d^	none	very low	58/17,285 (0.3%)	23/8813 (0.3%)	OR 1.03 (0.34 to 3.13)
Deep vein thrombosis	30,158 (5 non-randomised studies)	seriousᵃ	not serious	not serious	serious^d^	none	very low	175/20,533 (0.9%)	49/9625 (0.5%)	OR 0.71 (0.44 to 1.15)
Acute kidney injury	30,194 (5 non-randomised studies)	seriousᵃ	not serious	not serious	serious^d^	none	very low	623/20,569 (3.0%)	153/9625 (1.6%)	OR 0.85 (0.69 to 1.04)
90-day readmission	30,158 (5 non-randomised studies)	seriousᵃ	not serious	not serious	not serious	none	very low	1535/20,533 (7.5%)	462/9625 (4.8%)	OR 0.81 (0.69 to 0.94)

Reporting Bias

Reporting bias was not formally assessed due to fewer than 10 studies per outcome, consistent with Cochrane guidance [19]. No selective outcome reporting was detected where study protocols were available.

Discussions

This systematic review and meta-analysis found that GLP-1 RA use was associated with a statistically significant reduction in 90-day readmissions and short-term revision following THA, although the certainty of evidence was very low. Most other outcomes - including PJI, DVT, and AKI - showed non-significant trends favouring GLP-1 RAs. In contrast, outcomes such as wound dehiscence and long-term PPF slightly favoured the control.

The reduced risk in short-term revision may reflect improved glycaemic control as elevated HbA1c levels are a recognised risk factor for PJI, a significant cause of early revision [33,34]. GLP-1 RAs enhance insulin secretion and suppress glucagon, potentially reducing infection risk and subsequent revision [35]. Although the reduction in PJI did not reach significance, the direction of the pooled effect supports a plausible mechanism.

Weight loss may also contribute. Obesity is a known risk factor for revision and dislocation after THA [36]. In the STEP 5 trial, over 60% of semaglutide-treated patients achieved ≥10% weight loss at two years [18]. Xie et al. reported lower revision rates in patients who lost ≥10% weight after starting anti-obesity therapy [37]. Reduced adiposity may also lessen intraoperative difficulty and reduce operative times - factors associated with fewer readmissions [38].

Outcomes that slightly favoured controls - such as wound dehiscence and long-term PPF - are likely influenced by factors beyond GLP-1 RA action. Wound healing can be impaired by smoking-related vascular insufficiency, while PFF is linked to prosthesis instability and poor bone quality, which short-term metabolic therapy does not address [39,40]. Although GLP-1 RAs show favourable effects on bone turnover markers and trabecular structure in preclinical studies, human data remain inconclusive [41]. Reported effects on bone mineral density are inconsistent and appear agent-specific, with liraglutide showing potential benefit and exenatide yielding neutral or adverse results [42].

A recent review by Chan et al. assessed GLP-1 RA use and surgical complications following joint arthroplasty [43]. Whilst findings for PPF and long-term revision aligned with our review, they reported reduced PJI risk, which our review did not. Differences likely reflect methodology: Chan et al. combined hip and knee arthroplasty data without hip subgroup analysis. Our review also included two recent large studies -Levidy et al. and Verhey et al. - both of which provided additional PJI data [30,32].

Limitations of the Evidence

The evidence had several limitations. The use of administrative data introduced potential misclassification of outcomes. Outcome classification also varied - septic and aseptic revisions were rarely distinguished, limiting interpretability. Exposure windows also differed: Kim et al. required more than six months of GLP-1 RA use [29], whilst Magruder et al. required a prescription at the time of surgery [31]. Given that GLP-1 RAs cause peak weight loss at around 60 weeks and improve HbA1c within 12 weeks, short exposure may not have allowed for full therapeutic benefit [44].

Population heterogeneity limited comparability. Three studies included only DM patients [28,30,31], while others included obese or mixed cohorts [27,29,32], introducing potential confounding by indication, as GLP-1 RA dosing varies by use [18]. Baseline HbA1c and perioperative BMI were not reported, limiting adjustment for glycaemic control and obesity as confounders for infection-related outcomes. Only Magruder et al. reported agent-specific data [31]. Collectively, these limitations prevented subgroup analyses by GLP-1 RA type and indication.

Limitations of the Review Process

This review was limited to English-language publications from selected databases, introducing potential language or publication bias. Only retrospective observational studies met the inclusion criteria; no randomised controlled trials (RCTs) were identified, limiting the ability to infer causality. Outcomes such as LOS had to be narratively summarised due to inconsistent reporting, and others were excluded from meta-analysis due to sparse data, meaning the review may not reflect the full breadth of existing evidence.

Clinical Implications

Enhanced Recovery After Surgery (ERAS) protocols for THA emphasise preoperative optimisation of modifiable risk factors, including obesity and DM [45]. GLP-1 RAs may have a role in this setting due to their dual benefits in improving glycaemic control and promoting weight loss. These agents could be considered in patients unsuitable for bariatric surgery or where lifestyle interventions have been ineffective. However, current evidence is limited as aforementioned. Prospective trials are needed to define their role in ERAS pathways for THA. The present findings should be viewed as exploratory and interpreted within the context of low-certainty evidence.

## Conclusions

This is the first systematic review and meta-analysis to examine the association between GLP-1 RA use and outcomes following THA. While the findings suggest a reduction in 90-day readmissions and short-term revision rates, the very low certainty of evidence underscores the need for high-quality prospective research. GLP-1 RAs may represent a promising adjunct in perioperative optimisation strategies for patients undergoing THA, particularly those with obesity or diabetes. Future studies should include RCTs and adopt standardised reporting of key outcomes to validate and clarify the clinical utility of GLP-1 RAs in this setting.

## Appendices

Appendix A 

**Table 3:** Full electronic search strategy for each database as of 01/06/2025.

Database	Search Strategy
Medline (via PubMed)	("Arthroplasty, Replacement, Hip"[Mesh] OR "Hip Prosthesis"[Mesh] OR "Hip Arthroplasty, Total"[Mesh] OR "hip arthroplasty"[tiab] OR "hip replacement"[tiab] OR "total hip arthroplasty"[tiab] OR "THA"[tiab]) AND ("Glucagon-Like Peptide 1"[Mesh] OR "Glucagon-Like Peptide 1 Receptor"[Mesh] OR "GLP-1 receptor agonist"[tiab] OR "GLP-1"[tiab] OR "incretin mimetic"[tiab] OR "semaglutide"[tiab] OR "liraglutide"[tiab] OR "dulaglutide"[tiab] OR "exenatide"[tiab] OR "lixisenatide"[tiab]) AND ("Postoperative Complications"[Mesh] OR "Surgical Wound Infection"[Mesh] OR "readmission"[tiab] OR "revision"[tiab] OR "reoperation"[tiab] OR "infection"[tiab] OR "infections"[tiab] OR "periprosthetic fracture"[tiab] OR "periprosthetic joint infection"[tiab] OR "PJI"[tiab]OR "medical complications"[tiab])
Embase	('hip arthroplasty'/exp OR 'hip prosthesis'/exp OR 'total hip arthroplasty':ti,ab OR 'hip arthroplasty':ti,ab OR 'hip replacement':ti,ab OR THA:ti,ab) AND ('glucagon like peptide 1'/exp OR 'glucagon like peptide 1 receptor agonist'/exp OR 'glucagon like peptide 1 receptor':ti,ab OR 'glp-1 receptor agonist':ti,ab OR 'GLP-1':ti,ab OR 'incretin mimetic':ti,ab OR semaglutide:ti,ab OR liraglutide:ti,ab OR dulaglutide:ti,ab OR exenatide:ti,ab OR lixisenatide:ti,ab) AND ('postoperative complication'/exp OR 'surgical wound infection'/exp OR readmission:ti,ab OR revision:ti,ab OR reoperation:ti,ab OR infection:ti,ab OR infections:ti,ab OR 'periprosthetic fracture':ti,ab OR 'periprosthetic joint infection':ti,ab OR PJI:ti,ab OR 'medical complication':ti,ab)
Cochrane Central Register of Controlled Trials (CENTRAL)	(hip arthroplasty OR hip replacement OR total hip arthroplasty OR hip prosthesis OR THA) AND (GLP-1 OR GLP-1 receptor agonist OR glucagon like peptide 1 OR glucagon like peptide 1 receptor OR incretin mimetic OR semaglutide OR liraglutide OR dulaglutide OR exenatide OR lixisenatide) AND (postoperative complication OR surgical wound infection OR infection OR infections OR readmission OR revision OR reoperation OR periprosthetic fracture OR periprosthetic joint infection OR PJI OR medical complication)

Appendix B 

**Table 4:** ROBINS-I risk of bias assessments with justifications for each included study. ROBINS-I: Risk of Bias in Non-randomized Studies of Intervention; GLP-1: Glucagon-like peptide 1

Study	ROBINS-I domain							
	Confounding	Selection of participants	Classification of interventions	Deviations from intended interventions	Missing data	Measurement of outcomes	Selection of reported results	Overall
Buddhiraju 2024	Did not match for BMI as a continuous variable	Participants were selected based on pre-intervention characteristics, with selection unlikely to be influenced by outcome status	Included if prescription recorded from 1 year to 15 days pre-op; may reflect short past use rather than sustained exposure	Retrospective design with no evidence of differential deviations from the intended intervention between groups	No indication of missing outcome data; relevant endpoints appeared complete and consistently reported across groups	Outcomes were derived from administrative or electronic health records, with potential for misclassification	All prespecified outcomes and time points were reported; where available, protocols were reviewed. No evidence of selective reporting	Moderate
Heo 2024	Key confounders, such as diabetes severity (HbA1c) and BMI as a continuous variable, were not counted for		Exposure is defined as ≥3 fills in 6 months or ≥1 90-day fill pre-op, ensuring reliable perioperative exposure					Moderate
Kim 2024			Defined as ≥3 months pre- and post-op use, ensuring clear perioperative exposure		Although data were not missing, multiple outcomes were reported as <11, obscuring the exact values			Moderate
Levidy 2025	Propensity score matching included key confounders such as BMI (categorised) and HbA1c, reducing the risk of residual confounding		Included if prescription recorded 12 months prior to surgery; may reflect short past use rather than sustained exposure		No indication of missing outcome data; relevant endpoints appeared complete and consistently reported across groups			Moderate
Magruder 2023	Key confounders, such as diabetes severity (HbA1c) and BMI as continuous variables, were not counted for		Exposure is defined as having a GLP-1 prescription at surgery; duration unclear, raising some misclassification risk					Moderate
Verhey 2024			Exposure window (1 year pre- to 2 years post-op) included postoperative initiators, causing high misclassification risk					Moderate
